# Ultrasound viscoelastic imaging in the noninvasive quantitative assessment of chronic kidney disease

**DOI:** 10.1080/0886022X.2024.2407882

**Published:** 2024-09-30

**Authors:** Han Yuan, Qun Huang, Jing Wen, Yong Gao

**Affiliations:** a Department of Ultrasound, First Affiliated Hospital of Guangxi Medical University, Guangxi, China; b Department of Hematology and Rheumatology, First Affiliated Hospital of Guangxi Medical University, Guangxi, China

**Keywords:** Ultrasound viscoelastic imaging, chronic kidney disease, renal function, interstitial fibrosis, inflammation

## Abstract

**Background:**

This study aims to evaluate the clinical application value of ultrasound viscoelastic imaging in noninvasive quantitative assessment of chronic kidney disease (CKD).

**Methods:**

A total of 332 patients with CKD and 190 healthy adults as a control group were prospectively enrolled. Before kidney biopsy, ultrasound viscoelastic imaging was performed to measure the mean stiffness value (Emean), mean viscosity coefficient (Vmean), and mean dispersion coefficient (Dmean) of the renal. CKD patients were divided into three groups based on estimated glomerular filtration rate. The differences in clinic, pathology, ultrasound image parameters between the control and patient groups, or among different CKD groups were compared. The correlation between viscoelastic parameters and pathology were analyzed.

**Results:**

Emean, Vmean, and Dmean in the control group were less than the CKD group (*p* < 0.05). In the identification of CKD from control groups, the area under curve of Vmean, Dmean, Emean, and combining the three parameters is 0.90, 0.79, 0.69, 0.91, respectively. Dmean and Vmean were increased with the decline of renal function (*p* < 0.05). Vmean and Dmean were positively correlated with white blood cell, urea, serum creatinine, and uric acid (*p* < 0.05). Vmean is positively correlated with interstitial fibrosis and inflammatory cell infiltration grades (*p* < 0.001).

**Conclusions:**

Ultrasound viscoelastic imaging has advantages in noninvasive quantitative identification and evaluating renal function of CKD. Emean > 6.61 kPa, Vmean > 1.86 Pa·s, or Dmean > 7.51 m/s/kHz may suggest renal dysfunction. Combining Vmean, Dmean, and Emean can improve the efficiency of identifying CKD.

## Introduction

Recently, the incidence of chronic kidney disease (CKD) has been on the rise, with a global prevalence of 9.1% in 2017, becoming a threat to global health [1, 2]. It is predicted that by 2024, CKD will become the fifth leading cause of death globally [3]. Based on estimating the glomerular filtration rate (eGFR), CKD can be classified into five stages (G_1_–G_5_), G_1_: normal or mildly elevated renal function; G_2_: mild decreased renal function; G_3_: moderate decline in renal function; G_4_: severe renal decline; G_5_: stage of renal failure [4]. Prognosis varies from different CKD stages. Clinical interventions for early CKD patients can effectively prevent further damage to renal function [4]. With the prolongation of the disease course, renal function gradually deteriorates, and the risk of adverse outcomes increases. About 50% of patients with advanced CKD (G_4-5_) develop cardiovascular disease [5,6]. Patients with CKD beyond G_3_ stage have double the risk of atrial fibrillation and acute coronary syndrome, and the incidence of heart failure is also three times higher in patients with CKD beyond G_3_ stage compared to G_1_ stage[7]. Different stages of CKD require different treatment strategies. In the early stages of CKD (G_1-2_), it is necessary to identify the causes for precise treatment [4]. In the treatment of the G_3_ stage, during the compensatory period of the kidneys, the focus is on delaying the progression of the disease and controlling complications to reduce the risk of death [4]. In the G_4-5_ stage, it is necessary to evaluate the methods for kidney replacement therapy [4]. Therefore, the timely adjustment of the treatment regimen is helpful to delay the disease’s progression and improve the patient’s prognosis.

The eGFR based on serum creatinine (Scr) is widely used in the clinical staging of CKD due to its convenience and cost-effectiveness [4]. However, it is influenced by various factors, such as race, gender, age, and muscle mass [8, 9]. Glomerular disease is one of the causes of CKD. Renal interstitial fibrosis is an important indicator of renal function decline and prognosis [10–12]. Kidney biopsy, which is the gold standard for evaluating glomerular disease and renal fibrosis, is not the preferred method for real-time assessment of changes in CKD condition due to its invasive nature and corresponding complications, such as bleeding, infection, and arteriovenous fistula [13].

Functional magnetic resonance imaging (fMRI) can distinguish between different CKD stages, but it also has limitations, such as long scan time, respiratory movement interference, and magnetic susceptibility artifacts [14–16]. Computed tomography (CT) and intravenous urography (IVU) can provide information about kidney function while showing kidney morphology. However, the examination process requires using contrast agents, which can be nephrotoxic and unsuitable for patients with kidney dysfunction [17]. Nuclear medicine imaging can provide a relatively accurate evaluation of kidney function. Because of its radioactive elements and radiation, such imaging is inappropriate for long-term disease monitoring [18].

In comparison, ultrasounds can noninvasively and dynamically monitor kidney morphology and blood flow changes in real time without radiation and have good repeatability. Shear wave elastography (SWE) can quantitatively evaluate tissue elasticity and has advantages in studying glomerular diseases, renal fibrosis, renal damage, and prognosis [19–24]. However, some assessments of renal fibrosis and elastography have produced conflicting results [25]. In addition to anisotropy, the measurement of renal elasticity is also affected by blood perfusion and viscosity [26]. Biological soft tissues exhibit properties of both elasticity and viscosity, but in most ultrasound elastography currently used, only tissue elasticity is quantified, while tissue viscosity is often ignored.

A new ultrasound viscoelastic imaging method, which can simultaneously measure viscosity and stiffness of the tissue, has been recently used to evaluate the fibrosis and inflammatory necrosis of diffuse liver diseases [27–29]. It was found that elasticity was superior to viscosity in evaluating liver fibrosis, while viscosity was superior to elasticity in evaluating the degree of inflammation and necrosis [30, 31]. However, few studies have applied it to CKD. This study aimed to evaluate the clinical application of ultrasound viscoelastic imaging in the quantitative evaluation of the degrees of renal dysfunction in CKD patients.

## Materials and methods

### Patients

This prospective study was approved by the Ethics Committee (Approval number: 2023-E658-01). All enrolled subjects signed an informed consent form. We prospectively recruited 332 CKD patients were enrolled from June 2023 to November 2023. The inclusion criteria are as follows: (i) age ≥ 18 years; (ii) undergo a renal biopsy; and (iii) undergo an ultrasound and viscoelastic imaging examination within 3 days before the renal biopsy. The exclusion criteria are as follows: (i) the presence of concomitant renal diseases, as they can affect the measurements, including renal cysts and renal masses; (ii) renal parenchyma <1cm; (iii) individuals with renal artery stenosis or compression of the left renal vein; (iv) poor image quality; (v) contraindications for renal biopsy. Shear waves will attenuate during propagation, and most of the shear wave velocities are measured within 8 cm from the skin [32]. Therefore, individuals with a depth from renal cortex to skin surface exceeding 8 cm were also excluded from this study. The general information on the patients and their laboratory examination results were recorded. The eGFR was estimated based on the modified glomerular filtration rate estimating equation [33]:

(1) $$\begin{array}{c} e-\text{GFR }\left(\text{mL}/\text{min}/1.\text{73 }m^{2}\right)\;=175\times \text{serum creatinine }{\left(\text{mg}/\text{dL}\right)}^{-1.234}\\ \times \text{Age}{\left(y\right)}^{-0.179}\times 0.79\left(\text{if female}\right). \end{array}$$

According to eGFR, CKD patients were divided into three groups: normal or mild decline (eGFR ≥60 mL/min/1.73 m^2^), moderate decline (eGFR 30-59 mL/min/1.73m^2^), and severe decline (eGFR <30mL/min/1.73m^2^).

**Figure 1.**
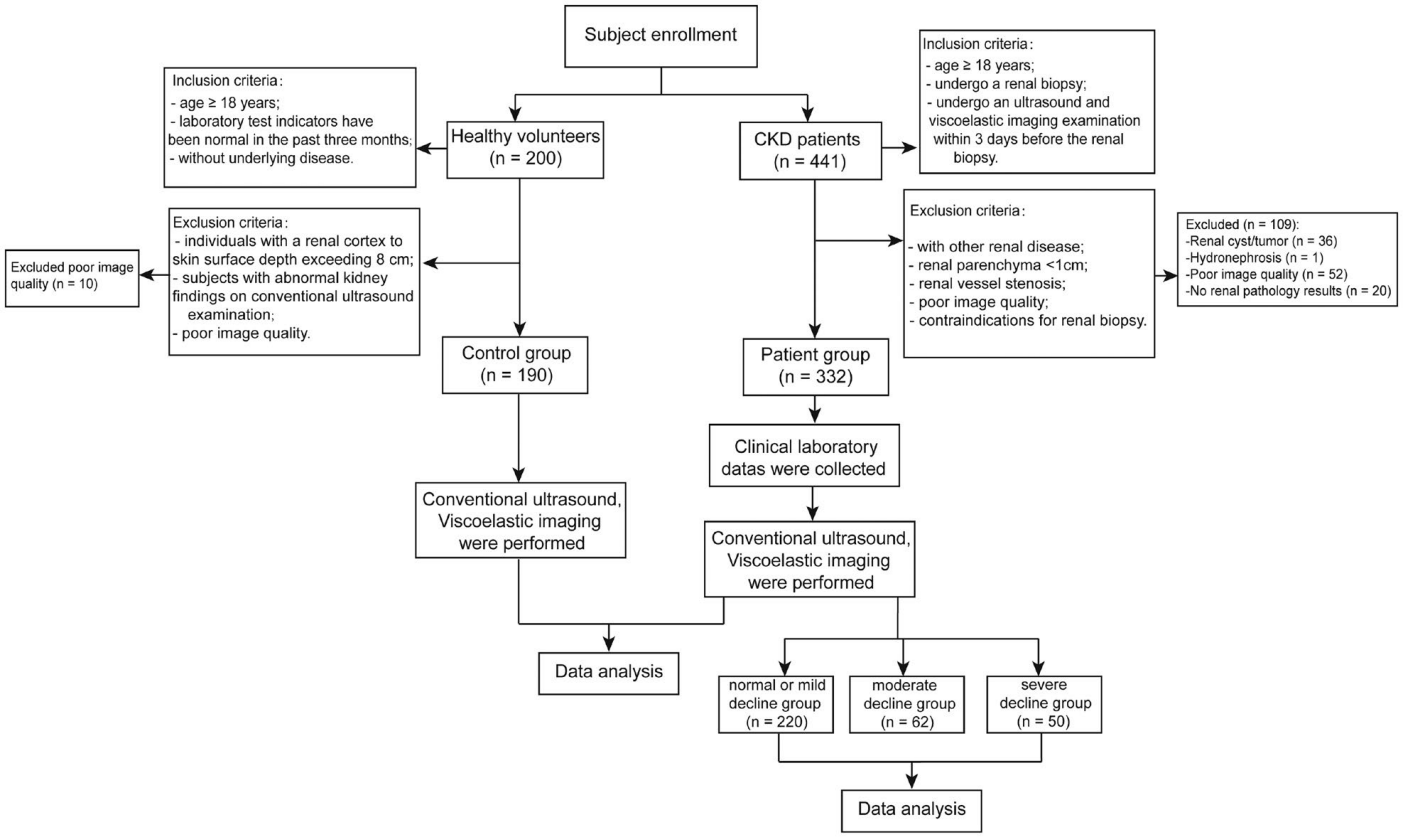
Research design and participants.

From June 2023 to November 2023, we recruited 190 healthy volunteers as the control group through questionnaires. The inclusion criteria are as follows: (i) age ≥ 18 years; (ii) laboratory test indicators have been normal in the past three months; (iii) without underlying disease such as kidney disease, hypertension or diabetes. The exclusion criteria are as follows: (i) individuals with a renal cortex to skin surface depth exceeding 8 cm; (ii) subjects with abnormal kidney findings on conventional ultrasound examination; (iii) poor image quality. Figure 1 demonstrates the process of this study.

### Conventional ultrasound and ultrasound viscoelastic imaging

A Mindray ultrasound system was used (Resona R9, Mindray Bio-Medical Electronics Co, China), equipped with a new elastic imaging software (4.1.0, Rev179255), a C5-1 transducer (Mindray Bio-Medical Electronics Co, China), with frequency range of 1–5MHz. Elastic imaging software allows for simultaneous quantification of tissue stiffness and viscosity. The viscosity coefficient (Vmean), which is related to tissue viscosity, is calculated using the Voigt model. The Voigt model employs more complex equations to explain velocity variations associated with shear wave frequency, and the formula is as follows:

(2) $$C=\sqrt{\frac{2(G^{2}+\omega^{2}\mu^{2})}{\rho (G+\sqrt{G^{2}+\omega^{2}\mu^{2}})}.}$$

where *C* is the shear wave phase speed (m/s), *G* is the renal tissue shear modulus (Pa), *ρ* is the media density, *µ* is the renal tissue shear viscosity (Pa·s), and *ω* is the angular frequency (rad/s) [34]. The dispersion coefficient (Dmean) uses a simplified linear formula, which is the slope of the fitting curve between propagation velocity and shear wave frequency change, as an indirect measurement parameter related to viscosity. Its formula is as follows:

(3) C=slope*f + a,

where *C* is the shear wave phase speed (m/s), *slope* is the dispersion slope coefficient, *f* is the frequency that changes during the propagation of the shear wave transverse wave (kHz), and *a* is a constant [26]. Mindray’s viscoelastic processing software utilizes single-frequency point technology, with a range of *f* from 200 to 1000 Hz. It collects velocities at 200 Hz, 400 Hz, 600 Hz, and 800 Hz for linear fitting. The ultrasound examinations were consistently conducted by an experienced sonographer (with 10 years of experience), who was unaware of the patients’ information. After urination, the patient was placed in a lateral position for routine renal ultrasound and elastography. The maximum long-axis section of the kidney was chosen for measuring the length, parenchymal thickness, and hemodynamic indices of renal arteries. After ensuring stable image quality, the viscoelastic imaging function was activated. During this process, the probe was kept vertical and stable on the skin to avoid applying additional pressure. The sample frame of approximately 2 × 2 cm was positioned on the parenchyma of the midportion of the kidney, beneath the renal capsule; the patient was instructed to hold their breath for 6 s. The image was frozen after it filled with color. Subsequently, the quantitative sampling box (Qbox) was positioned in the renal parenchyma of the sampling frame below the renal envelope. The Qbox dimensions were configured to 1 cm. The viscoelastic imaging system will automatically calculate Young’s modulus, viscosity coefficient and dispersion coefficient in Qbox. Emean represents the average elasticity of the tissue in QBox with kPa, which is obtained from the formula [35]:

(4) $$E=3\rho V^{2},$$

*E* represents the stiffness of the tissue, *ρ* represents the density, and *V* is the estimated shear wave velocity. Vmean represents the average viscosity coefficient with Pa·s, and Dmean represents the average dispersion coefficient with m/s/kHz. The above procedure was repeated five times to improve accuracy, obtaining the average value.

The viscoelastic imaging can simultaneously provide SWE imaging, reliability map, and viscosity or dispersion imaging (Supplementary Figure 1). Presently, there lacks a standardized quality control criterion for renal elastography [36, 37]. Therefore, we have used published studies to establish the following quality control standards for this research: (i) the image should exhibit uniform color saturation and possess a moving stability index (M-STB) rating of three stars or higher; (ii) the image should demonstrate a reliability index of 90% or above; and (iii) the interquartile range (IQR)-to-median ratio should not exceed 30% [38–43].

In the healthy control group, we selected 50 subjects to conduct intra-group and inter-group consistency tests. The viscoelastic parameters of the left kidney were measured by two sonographers (sonographer A and sonographer B) with 10 years of experience in ultrasound for inter-observer consistency testing, and the consistency between observers was demonstrated using Bland-Altman plots. One week later, sonographer A re-measured the viscoelastic parameters of the left kidney of these 50 subjects to conduct intra-observer consistency testing. Ultimately, the viscoelastic data measured by sonographer A were used for the subsequent statistical analysis. Since CKD patients usually choose the left kidney for puncture biopsy, we selected the left kidney ultrasound and elasticity measurement parameters for comparative analysis between the control and case groups.

### Kidney biopsy and histopathology

A kidney biopsy was conducted within 3 days following elastography, with ultrasound guidance and an 18 G needle, targeting the lower pole parenchyma of the left kidney. The renal tissue was collected, fixed in 10% formalin, and subsequently sent for histopathological research. Paraffin slices were analyzed using hematoxylin and eosin, periodic acid–Schiff, and Masson trichrome staining. According to Banff semi-quantitative scoring criteria, interstitial fibrosis and inflammatory cell infiltration were rated into 4 grades according to the percentage of the lesion in the cortex area: 0(none);1+(<25%); 2+(25%-50%); and 3+(>50%) [44].

### Statistical analysis

Statistical analysis was performed using SPSS (version 26.0; SPSS) and MedCalc software (version 20.0; MedCalc). We used the Shapiro-Wilk method to test the normality of the data. Continuous variables that follow a normal distribution or approximate normal distribution are expressed as mean ± standard deviation (SD), and the comparison between groups was performed using the t-test. Non-normally data distributed as median and interquartile range (IQR), and Mann-Whitney U was used for comparison between the two groups. Categorical variables were presented as numbers or percentages and were compared using the chi-square test. One-way ANOVA was used to compare the continuous variables between multiple groups. The Kruskal-Wallis test was used to compare between groups of rank data. Pearson or Spearman correlation coefficients were used to indicate the relationship between variables. The diagnostic performance of viscoelastic parameters was compared using ROC curves; *p* < 0.05 was considered to indicate statistically significant differences.

## Results

### Cohort characteristics

A total of 441 patients were continuously enrolled from June 2023 to November 2023. We excluded 109 patients based on the exclusion criteria (36 patients with renal cysts/tumors, one patient with hydronephrosis, 52 patients had poor image quality, 20 patients with no pathology results). A total of 200 healthy volunteers were recruited, excluding 10 with poor image quality. Finally, 190 healthy adults(mean age, 46.88 ± 13.41 years; 110 females, 80 males)and 332 CKD patients (mean age, 42.3 ± 15.4 years; 185 females, 147 males) were included in this study. Demographic characteristics of participants are shown in Table 1. Groups based on eGFR are as follows: normal or mild decline group (*n* = 220), moderate decline group (*n* = 62), severe decline group (*n* = 50).

**Table 1.** Demographic data of study subjects.

	Age(years)	Sex		BMI (kg/m^2^)
		male	female	
Control group (*n* = 190)	46.88 ± 13.41	80	110	23.03 ± 3.17
CKD group (*n* = 332)	42.33 ± 15.44	147	185	23.25 ± 3.99
*p*	< 0.001	0.63		0.486

CKD: chronic kidney disease; BMI: body mass index.

### Conventional ultrasound and viscoelastic imaging

The comparison of conventional ultrasound and viscoelastic parameters of the kidney in the control group is shown in Table 2. Emean, Vmean and Dmean showed no statistical significance between the left and right kidneys (*p* > 0.05). In addition, to explore the influence of baseline data on ultrasound viscoelastic parameters, we conducted univariate logistic regression based on the baseline data of the healthy control group (Supplementary Table 1). Conventional ultrasound parameters between the control and CKD groups are shown in Table 3. The parameters of conventional ultrasound and viscoelasticity in different CKD groups are shown in Table 4. The peak systolic velocity (PSV) of the renal aorta, renal segmentary artery, and interlobar artery decreases with the decline of renal function (*p* < 0.05). There was no significant difference in Emean in different CKD groups (*p* > 0.05). Vmean and Dmean increased with the decrease of renal function (*p* < 0.05).

**Table 2.** Study data of the healthy control group.

Characteristic	Left kidney	Right kidney	*p*
Conventional ultrasound			
Kidney length (cm)	10.20 ± 0.89	9.90 ± 0.61	< 0.001
Renal parenchymal thickness(cm)	1.79 ± 0.22	1.70 ± 0.25	< 0.001
PSV of renal artery(cm/s)	66.66 ± 12.88	66.51 ± 12.63	0.854
PSV of segmental arteries(cm/s)	44.47 ± 6.48	45.93 ± 7.0	0.003
PSV of interlobar arteries(cm/s)	27.34 (23.81-30.95)	27.61 (24.64-30.72)	0.83
RI of renal artery	0.61 ± 0.05	0.61 ± 0.05	0.42
RI of segmental arteries	0.57 (0.54-0.60)	0.57 (0.54-0.62)	0.056
RI of interlobar arteries	0.52 ± 0.05	0.53 ± 0.05	0.001
Emean (kPa)	5.81 ± 1.1	5.85 ± 0.97	0.567
Vmean (Pa·s)	1.73 ± 0.13	1.73 ± 0.11	0.735
Dmean (m/s/kHz)	6.90 ± 0.70	6.85 ± 0.63	0.26

PSV: peak systolic velocity; RI: resistive index; Emean: the mean of stiffness value; Vmean: the mean of viscosity coefficient; Dmean: the mean of dispersion coefficient.

**Table 3.** Conventional ultrasound and viscoelasticity data of study subjects.

Characteristic	Control group (n = 190)	CKD group (n = 332)	*p*
Conventional ultrasound			
Kidney length (cm)	10.20 ± 0.89	10.15 ± 0.90	0.50
Renal parenchymal thickness(cm)	1.79 ± 0.22	1.63 ± 0.26	< 0.001
PSV of renal artery(cm/s)	66.66 ± 12.88	60.37 ± 13.83	< 0.001
PSV of segmental arteries(cm/s)	44.47 ± 6.48	42.58 ± 8.73	0.005
PSV of interlobar arteries(cm/s)	27.34 (23.81-30.95)	25.22 (21.42-29.95)	< 0.001
RI of renal artery	0.61 ± 0.05	0.61 ± 0.07	0.456
RI of segmental arteries	0.57 (0.54-0.60)	057 (0.53-0.61)	0.691
RI of interlobar arteries	0.52 ± 0.05	0.51 ± 0.06	0.18
Viscoelastic imaging			
Emean (kPa)	5.81 ± 1.1	6.84 ± 1.72	< 0.001
Vmean (Pa·s)	1.73 ± 0.13	2.08 ± 0.29	< 0.001
Dmean (m/s/kHz)	6.90 ± 0.70	7.79 ± 0.92	< 0.001

PSV: peak systolic velocity; RI: resistive index; Emean: the mean of stiffness value; Vmean: the mean of viscosity coefficient; Dmean: the mean of dispersion coefficient.

**Table 4.** Comparison of conventional ultrasound and viscoelasticity data in different CKD groups.

	Normal or mild decline group (*n* = 220)	Moderate decline group (*n* = 62)	Severe decline group (*n* = 50)	*p*
Conventional ultrasound				
Kidney length (cm)	10.29 ± 0.80	9.84 ± 0.99^a^	9.89 ± 1.06^a^	< 0.001
Parenchymal thickness(cm)	1.68 ± 0.24	1.57 ± 0.27^a^	1.53 ± 0.26^a b^	< 0.001
PSV of renal artery (cm/s)	64.42 ± 12.06	56.0 ± 12.29^a^	47.98 ± 14.16^a b^	0.002
PSV of segmental arteries (cm/s)	44.56 ± 7.34	41.44 ± 9.22^a^	35.25 ± 9.36^a b^	< 0.001
PSV of interlobar arteries (cm/s)	27.36 ± 5.95	24.76 ± 4.73^a^	21.39 ± 4.61^a b^	< 0.001
RI of renal artery	0.59 ± 0.06	0.63 ± 0.05^a^	0.66 ± 0.07^a b^	< 0.001
RI of segmental arteries	0.56 ± 0.06	0.59 ± 0.06^a^	0.61 ± 0.06^a^	< 0.001
RI of interlobar arteries	0.51 ± 0.06	0.52 ± 0.06	0.51 ± 0.06	0.793
RI of renal artery	0.59 ± 0.06	0.63 ± 0.05^a^	0.66 ± 0.07^a b^	< 0.001
Viscoelastic imaging				
Emean (kPa)	6.91 ± 1.64	6.82 ± 1.79	6.55 ± 1.97	0.409
Vmean (Pa·s)	2.01 ± 0.23	2.10 ± 0.24^a^	2.36 ± 0.42^a b^	< 0.001
Dmean (m/s/kHz)	7.60 ± 0.85	7.93 ± 0.87^a^	8.44 ± 0.98^a b^	< 0.001

^a^ *p* < 0.05 vs the normal or mild decline group;.

^b^ *p* < 0.05 vs the moderate decline group. Emean the mean of stiffness value, Vmean the mean of viscosity coefficient, Dmean the mean of dispersion coefficient. PSV peak systolic velocity, RI resistive index.

### Intergroup and intragroup consistency analysis

The intra-observer consistency of Emean, Vmean, and Dmean were 0.96 (95%CI: 0.93–0.98), 0.97 (95% CI: 0.93–0.98), and 0.97 (95% CI: 0.94–0.98), respectively. The inter-observer consistency of Emean, Vmean, and Dmean were 0.77 (95% CI: 0.63–0.86), 0.90 (95%CI: 0.73–0.95), and 0.94 (95% CI: 0.85–0.97), respectively. Bland–Altman plot (Supplementary Figure 2) showed the inter-observer variability. The intra-observer and inter-observer consistency indicated that the viscoelastic measurement of the kidney had good reproducibility.

### Diagnostic performance

Viscoelastic parameters showed differences between the healthy control group and the patient group, and we combined the viscoelastic parameters to construct a combined model. The performance of viscoelastic parameters and the combined model in the prediction of CKD is shown in Table 5. Among the viscoelastic parameters, Vmean exhibits higher performance in predicting CKD (AUC 0.90), and the Delong test shows that the combined model incorporating all three viscoelastic parameters achieves the best predictive performance (AUC 0.91). The comparison of clinical and pathological parameters in different CKD groups and the correlation analysis with viscosity parameters are shown in Table 6. Representative cases are shown in Figures 2–3.

Figure 2.A 59-year-old female CKD patient with normal renal function of CKD. Pathological indicated minimal change disease (MCD), interstitial fibrosis (0), and inflammatory cell infiltration (1+).(A)The mean stiffness measured by shear wave elasticity is 4.06kPa. The mean viscosity coefficient measured by viscoelasticity is 1.76 Pa·s. (B)The mean dispersion coefficient of viscoelastic measurement is 7.42m/s/kHz. (C)HE staining, PASM staining and MASSON staining of renal pathology.

**Figure:**
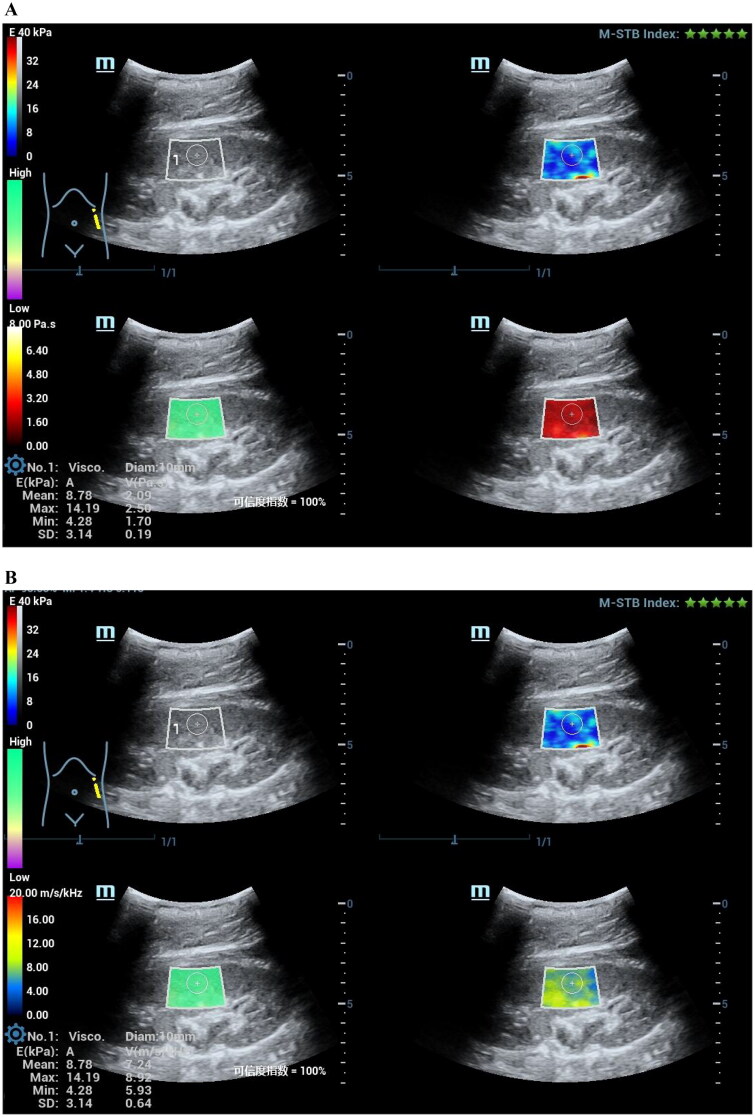

**Figure:**
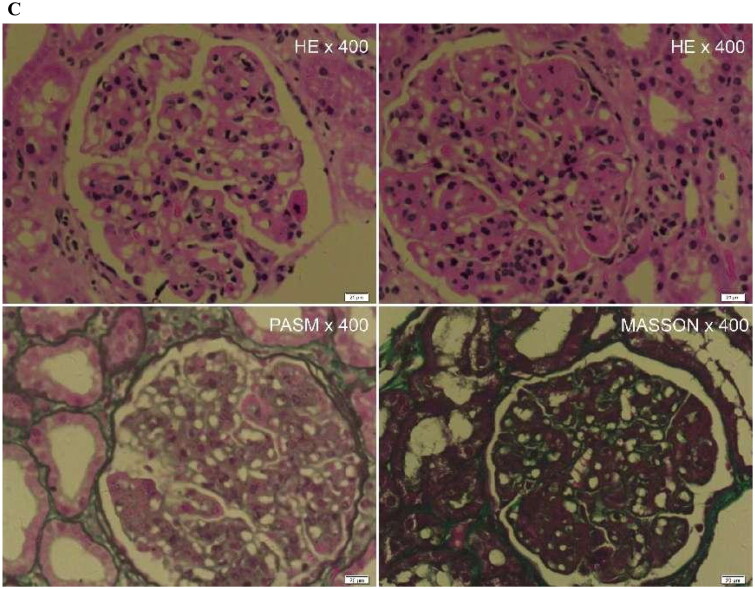

Figure 3.A 50-year-old female patient with severe decline renal function of CKD. Pathological indicated lupus nephritis (LN), interstitial fibrosis (2+), and inflammatory cell infiltration (2+).(A)The mean stiffness measured by shear wave elasticity is 5.56kPa. The mean viscosity coefficient measured by viscoelasticity is 3.70 Pa·s. (B)The mean dispersion coefficient of viscoelastic measurement is 9.99m/s/kHz. (C)HE staining, PASM staining, and MASSON staining of renal pathology.

**Figure:**
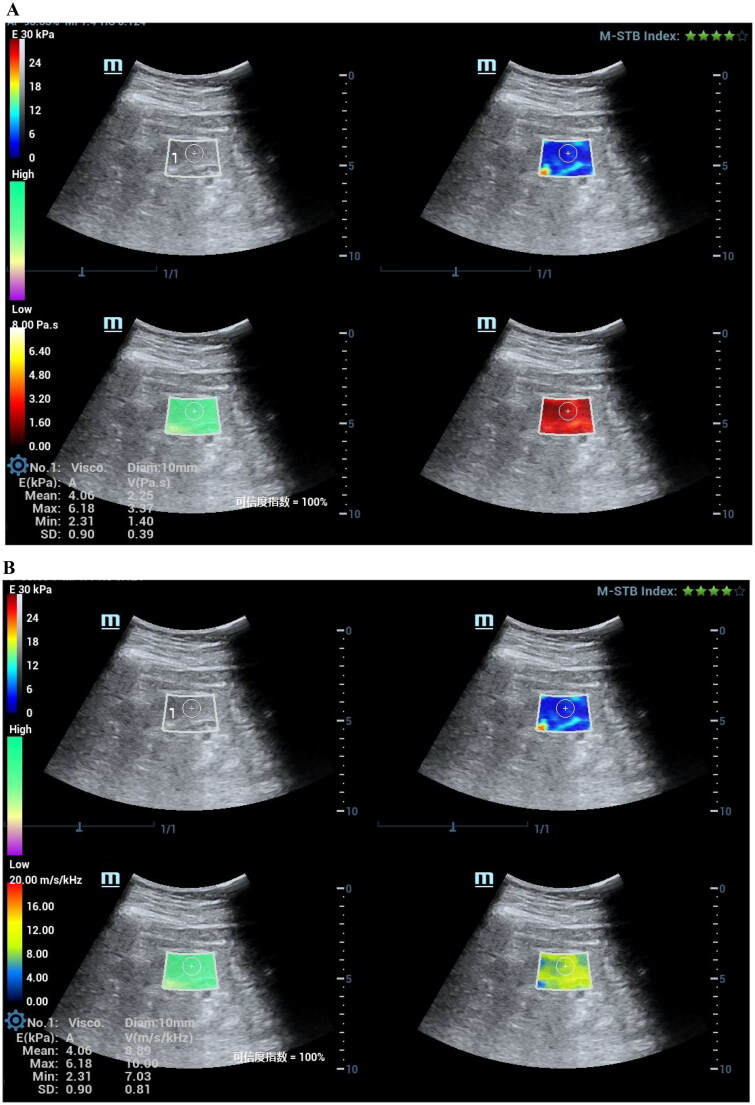

**Figure:**
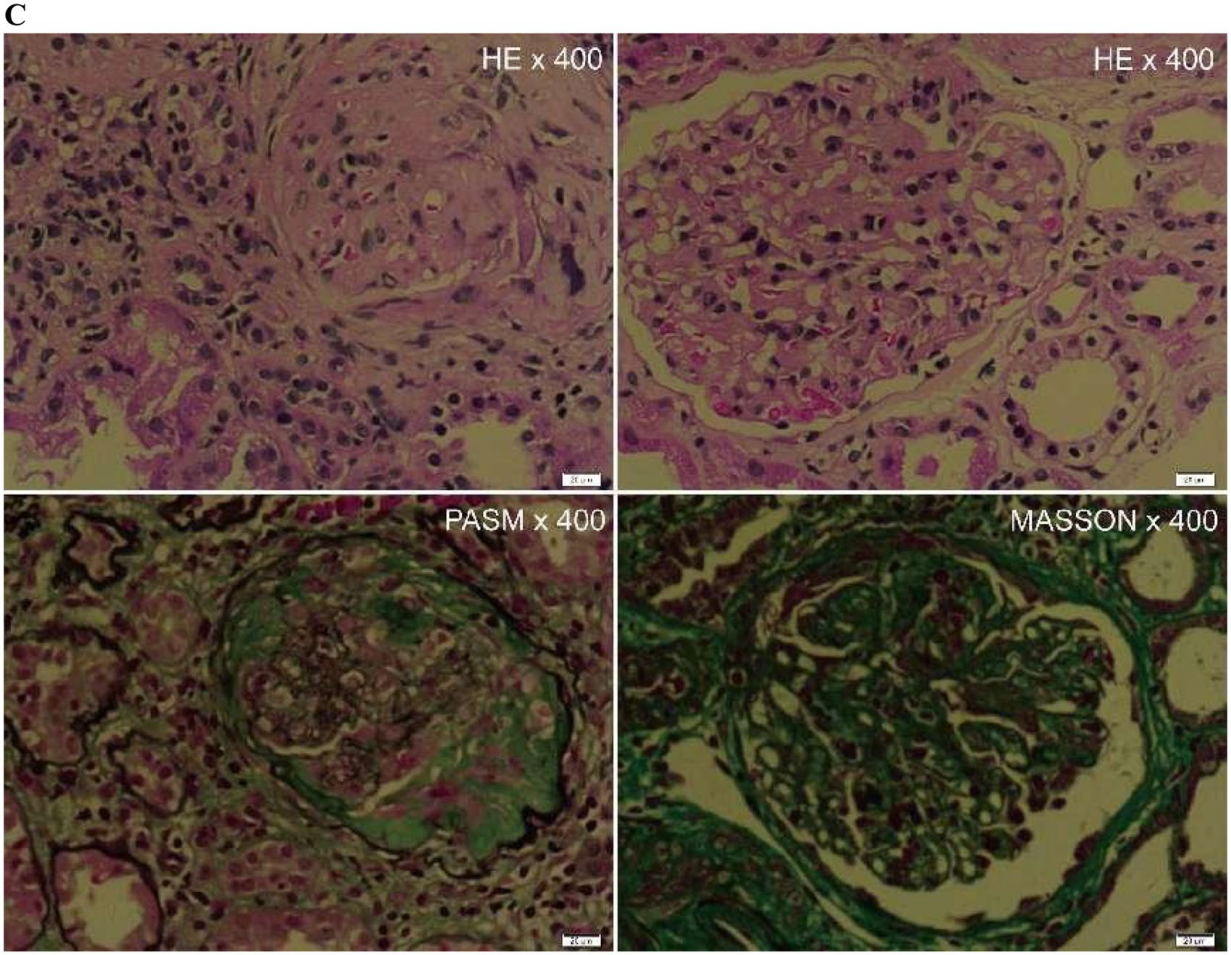

**Table 5.** The performance of viscoelastic parameters in evaluation renal function of CKD.

	AUC	95% CI	Sensitivity	Specificity	cutoff value
Emean(kPa)	0.69	0.65-0.73	0.52	0.79	> 6.61
Dmean(m/s/kHz)	0.79^a^	0.75-0.82	0.62	0.83	> 7.51
Vmean(Pa·s)	0.90^a^^b^	0.87-0.93	0.77	0.88	> 1.86
Combined model	0.91^a^^bc^	0.89-0.94	0.84	0.85	> 0.59

^a^ Delong test (1988), compared to Emean, *p* < 0.05. ^b^Delong test (1988), compared to Dmean, *p* < 0.05, ^c^Delong test (1988), compared to Vmean, *p* < 0.05.

**Table 6.** The clinicopathological parameters of the 332 CKD patients.

	Normal or mild decline group (*n* = 220)	Moderate decline group (*n* = 62)	Severe decline group (*n* = 50)	*p*	Pearson’s/Spearman’s rho (*p* value)		
					Vmean (Pa·s)	Dmean (m/s/kHz)	Emean (kPa)
WBC (10^9/L)	7.20 ± 2.96	7.83 ± 3.11	8.50 ± 2.85^a^	0.014	0.16 (0.003)	0.17 (0.002)	0.15 (0.78)
RBC (10^12/L)	4.21 ± 0.88	3.99 ± 1.05	3.72 ± 1.08^a^	0.003	−0.05 (0.351)	−0.10 (0.084)	0.14 (0.011)
HGB (g/L)	116.28 ± 25.51	108.76 ± 26.84^a^	98.84 ± 26.95^a^^^b^^	< 0.001	−0.04 (0.473)	−0.07 (0.383)	0.104 (0.060)
UREA (mmol/L)	5.35 ± 2.05	10.38 ± 8.05^a^	17.67 ± 8.86^a^^b^	< 0.001	0.28 (< 0.001)	0.24 (< 0.001)	−0.023 (0.673)
Scr (umol/L)	74.45 ± 2.43	143.74 ± 29.20^a^	369.32 ± 184.84^a^^b^	< 0.001	0.37 (< 0.001)	0.32 (< 0.001)	−0.052 (0.347)
UA (umol/L)	371.63 ± 108.46	436.44 ± 146.65^a^	457.86 ± 136.17^a^	< 0.001	0.17 (0.002)	0.15 (0.007)	−0.013 (0.816)
eGFR(mL/min/1.73m^2^)	109.66 ± 44.33	45.78 ± 13.28^a^	17.31 ± 7.10^a^^b^	< 0.001	−0.35 (< 0.001)	−0.31 (< 0.001)	0.043 (0.432)
IF(grade)				< 0.001	0.18 (0.001)	0.10 (0.062)	0.000 (0.999)
0	134 (60.91%)	20 (32.26%)	3 (6.0%)	/	/	/	/
1	77 (35.0%)	22 (35.48%)	12 (24.0%)	/	/	/	/
2	9 (4.09%)	13 (20.97%)	24 (48.0%)	/	/	/	/
3	/	7 (11.29%)	11 (22.0%)	/	/	/	/
ICI(grade)				< 0.001	0.14 (0.014)	0.06 (0.253)	−0.042 (0.447)
0	114 (51.82%)	10 (16.13%)	4 (8.0%)	/	/	/	/
1	92 (41.82%)	36 (58.06%)	18 (36.0%)	/	/	/	/
2	14 (6.36%)	11 (17.74%)	22 (44.0%)	/	/	/	/
3	/	5 (8.07%)	6 (12.0%)	/	/	/	/

^a^ *p* < 0.05 vs the normal or mild decline group; ^b^*p* < 0.05 vs the moderate decline group. WBC white blood cell, RBC: red blood cell; HGB: hemoglobin; Scr: serum creatinine; UA: uric acid; IF: interstitial fibrosis; ICI: inflammatory cell infiltration.

## Discussion

This study investigated the application value of a novel elastography technique (ultrasound viscoelasticity imaging) in CKD. Our results indicate that the viscosity coefficient and dispersion coefficient that reflect tissue viscosity can not only differentiate between normal kidneys and those of CKD patients, but also between CKD patients with different degrees of renal dysfunction. If the value of Emean > 6.61 kPa, or the value of Vmean > 1.86 Pa·s, or the value of Dmean > 7.51 m/s/kHz, it may indicate renal dysfunction in the patient. When combining the three parameters, the AUC increases to 0.91, and by applying a probability threshold of 0.59 as the cutoff value, the sensitivity improves to 0.84. The higher the values of Vmean and Dmean, the higher the degree of possible renal dysfunction.

In this study, there were differences in the conventional ultrasound measurement parameters of kidney parenchymal thickness and the PSV in kidney artery between healthy individuals and patients. As the stage of CKD progressed, the length of the kidney, the thickness of the kidney parenchyma, and the PSV in kidney artery all showed a decreasing trend, while the resistive index showed an increasing trend. This is consistent with the pathological changes of CKD, that is, with the progression of CKD stages, there is progressive damage to nephrons, renal interstitial fibrosis, reduction in kidney volume, disruption of renal microcirculation, and decreased blood flow[45]. However, in this study, we found that the variation range in conventional ultrasound measurement parameters between CKD patients and the healthy control group, as well as among different CKD stages, were small, and overall remained within the normal reference range. This may be related to the fact that more patients in G_1_-G_2_ stages were included in this study, while fewer patients in G_3_-G_5_ stages were enrolled. In the early stages of CKD (G_1_-G_2_), the kidney has a strong compensatory function, and the structural changes in the kidney are subtle, making it difficult for conventional ultrasound to detect. Therefore, it is impossible to accurately identify CKD patients in the early stage and make an accurate assessment of the changes in renal function of CKD patients only through routine ultrasound examination.

In our current study, the baseline data indicated that the age of the healthy controls was older than that of the CKD patient group (*p* < 0.001), suggesting that age might be a confounding factor affecting the viscoelastic parameters between the two groups. To further investigate, we performed a univariate logistic regression analysis with viscoelastic parameters as the outcome variables and age as the independent variable. The results showed that age was not a factor influencing Emean, Vmean, and Dmean (*p* = 0.789, *p* = 0.554, and *p* = 0.39, respectively, Supplementary Table 1).

In the study, Emean, Vmean, and Dmean differ between the healthy control group and CKD patients (*p* < 0.05). Some studies have shown that ultrasound elastography can reflect the degree of renal fibrosis by measuring the stiffness [46, 47]. However, in this study, Emean did not differ significantly between different CKD groups, which reflects tissue stiffness. Wang et al. found that kidney stiffness was not predictive of CKD stage, which was consistent with our findings [48]. Sasaki et al. showed that renal stiffness was unrelated to kidney function [49]. In addition, Wu et al. also found that SWE was of low value in the staging of CKD [50]. The reason may be that the decrease in blood flow and perfusion pressure may decrease shear wave propagation velocity, indirectly resulting in a decrease in the stiffness value measured by SWE [51–53]. This is consistent with our data results, as CKD progresses, the PSV in the renal artery decreases (*p* < 0.05). The reason may be that with the occurrence and development of renal fibrosis, renal microcirculation is damaged, leading to a decrease in renal blood flow [54]. Changes in renal blood flow and perfusion pressure may offset some of the changes caused by fibrosis-induced stiffness. Therefore, applying elastic parameters that reflect stiffness cannot accurately evaluate changes in renal function in CKD patients.

In our study, Vmean and Dmean, which reflect tissue viscosity, showed differences among groups with different CKD groups (*p* < 0.05). With the decline of renal function, tissue viscosity tended to increase. Renal interstitial fibrosis is a common pathological feature of CKD, which is closely related to renal dysfunction [11]. At the same time, the sustained stimulation of renal interstitial inflammation can also lead to impaired glomerular function and renal fibrosis. The degree of inflammation is closely related to the deterioration of renal function and the degree of renal fibrosis [55]. Therefore, renal interstitial fibrosis, immune infiltration, and inflammatory response play a crucial role in the occurrence and development of CKD. Studies have found that viscosity is related to tissue inflammation and necrosis [27]. Viscosity is an important factor affecting the measurement of elasticity [26]. In our study, there is an increase in levels of inflammatory cell infiltration and interstitial fibrosis with CKD progression, which is consistent with the pathological changes that occur during CKD progression. The viscosity coefficient Vmean was positively correlated with the levels of inflammatory cell infiltration and interstitial fibrosis, which explains well the observed difference in viscosity coefficients among different groups ofCKD, as well as the tendency for viscosity coefficients to increase as renal function decreases. This may also be another reason why only using elastic parameters, which reflect renal stiffness, cannot accurately evaluate changes in the renal function of CKD patients. The AUC of Vmean and Dmean were more effective in predicting CKD than Emean (AUC: 0.90, 0.79, 0.69, respectively). However, the viscoelastic parameters were not sensitive in predicting CKD, which might be attributed to the fact that most of the subjects in this study were in G_1_-G_2_ stage, with reversible damage to nephrons, mild renal fibrosis, and mild inflammatory infiltration. When we combine the viscoelastic parameters, the AUC and sensitivity increases to 0.91 and 0.84, respectively. This indicates that compared with the traditional elastic Young’s modulus values, the new indicators of tissue viscosity (Vmean and Dmean) have advantages in differentiating CKD and different renal function. By using viscosity measurement parameters as evaluation indicators, the changes in renal function of CKD patients can be more sensitively monitored, providing a more accurate and sensitive tool for observing and evaluating the changes of renal function of CKD patients in the future.

The current study has some limitations. First, our research subjects were CKD patients who underwent renal biopsy, and we excluded those with renal parenchyma thickness < 1 cm and skin-to-renal parenchyma distance > 8 cm. This introduced a selection bias. In future studies, we will expand the scope of research subjects to further explore the application of viscoelastic imaging technology in CKD. Second, the current study did not undertake pathological classification within the included disease groups. Consequently, the applicability of elastography imaging across different pathological types remains unknown. Further investigation is required to assess the potential utility of elastography imaging in distinguishing among various pathological classifications. Additionally, as this was a single-center prospective study, there may be potential for selection bias. In the future, multi-center studies are needed.

## Conclusions

In conclusion, this study indicates that viscoelastic imaging has potential application value in evaluating chronic kidney disease. The new viscoelastic imaging parameters can more comprehensively evaluate the renal tissue changes in CKD. Emean > 6.61 kPa, Vmean > 1.86 Pa·s, or Dmean > 7.51 m/s/kHz may suggest renal dysfunction. Higher Vmean and Dmean values indicate greater dysfunction. Combining Vmean, Dmean, and Emean can effectively improve the efficiency of identifying CKD. In the clinical diagnosis and treatment of CKD, viscoelastic imaging is expected to become a new examination tool and provide more valuable reference information for dynamic assessment and monitoring of renal function changes.

## Supplementary Material

Supplemental Material

## Data Availability

The data that support the findings of this study are available from the corresponding author upon reasonable request.
