# Supplementary material for: Ultrasound viscoelastic imaging in the noninvasive quantitative assessment of chronic kidney disease
Source: Ren Fail. 2024 Sep 30;46(2):2407882. doi: 10.1080/0886022X.2024.2407882 (PMC11443565; doi:10.1080/0886022X.2024.2407882)
Supplement: Supplemental Material [file IRNF_A_2407882_SM9022.docx]

**Supplement materials**

**Supplementary table 1 Univariate analysis of factors affecting viscoelastic parameters**

| Characteristic | Emean | |  | Vmean | |  | Dmean | |
| --- | --- | --- | --- | --- | --- | --- | --- | --- |
|  | β | *p* |  | β | *p* |  | β | *p* |
| age | -0.002 | 0.789 |  | < 0.001 | 0.554 |  | 0.003 | 0.39 |
| Sex | -0.119 | 0.465 |  | 0.052 | 0.007 |  | 0.466 | < 0.001 |
| BMI | -0.05 | 0.047 |  | 0.008 | 0.011 |  | 0.056 | < 0.001 |

**Supplementary figure 1**

**
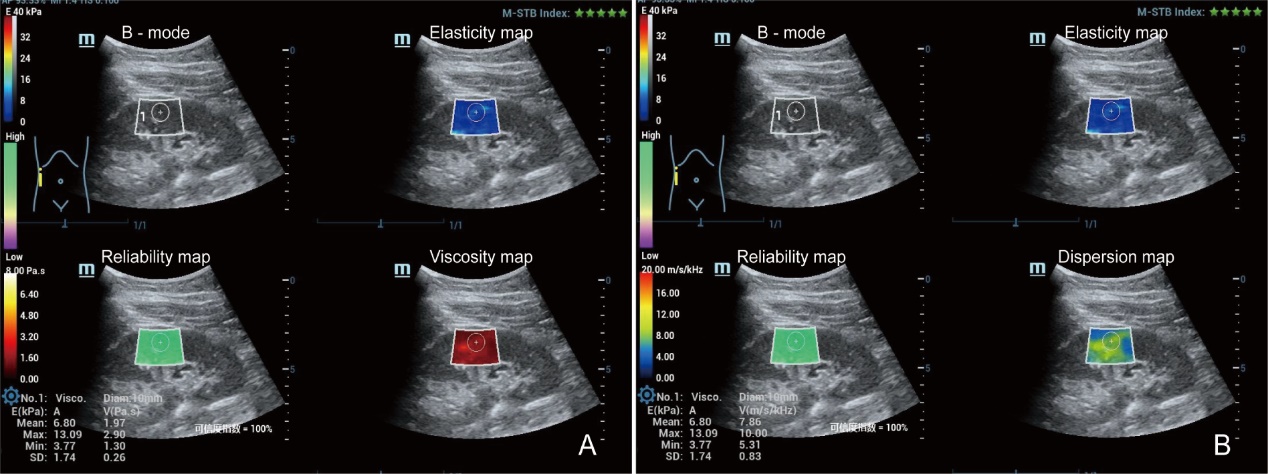
**

**Supplementary** **figure 1** Schematic diagram of viscoelastic imaging measurement.

After activating the viscoelastic imaging function, grayscale images (B - mode), elasticity images, reliability maps, viscosity maps, and dispersion maps are displayed simultaneously. We place the sampling box in the renal parenchyma at the middle of the kidney, and position the Qbox in the center of the sampling box, close to the renal capsule, for measurement. (A) Viscosity imaging. (B)  Dispersion slope imaging.

**Supplementary figure 2**


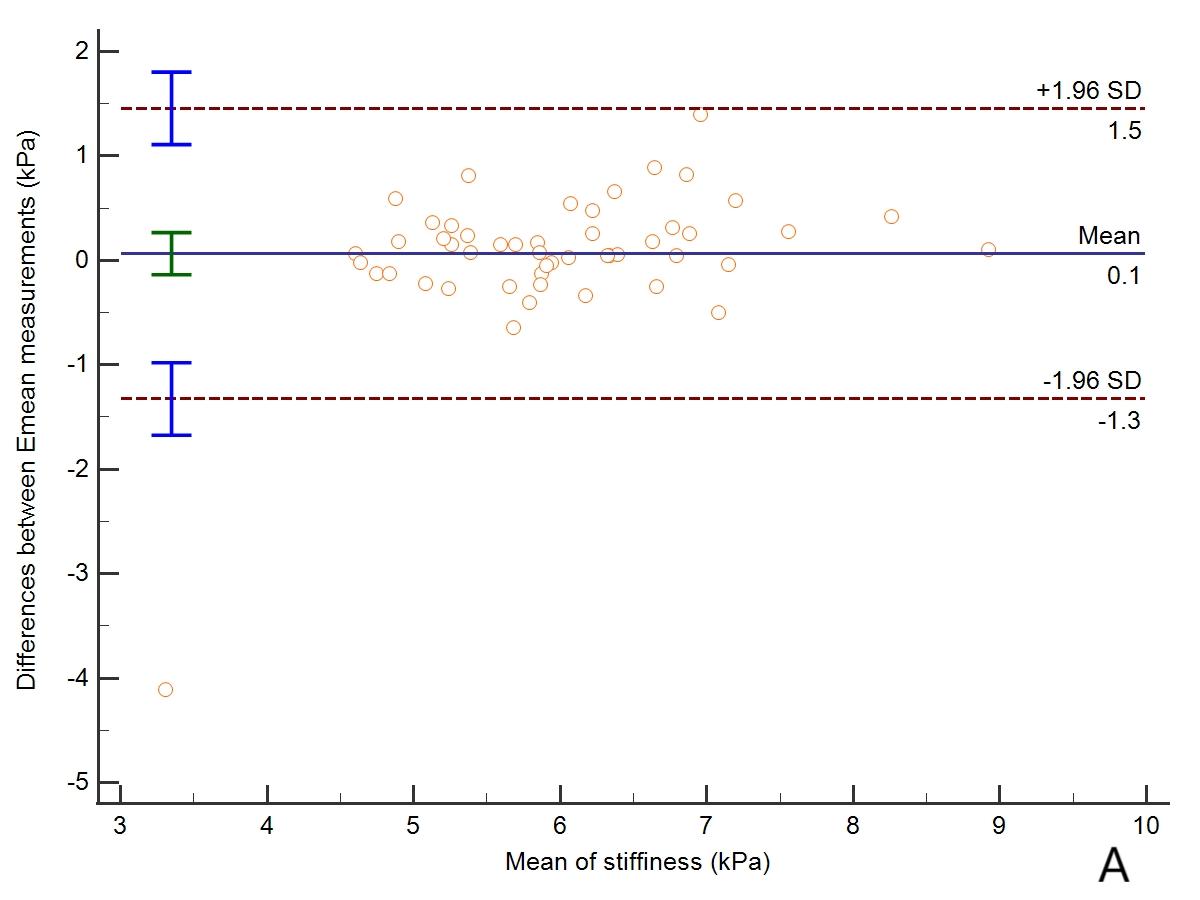


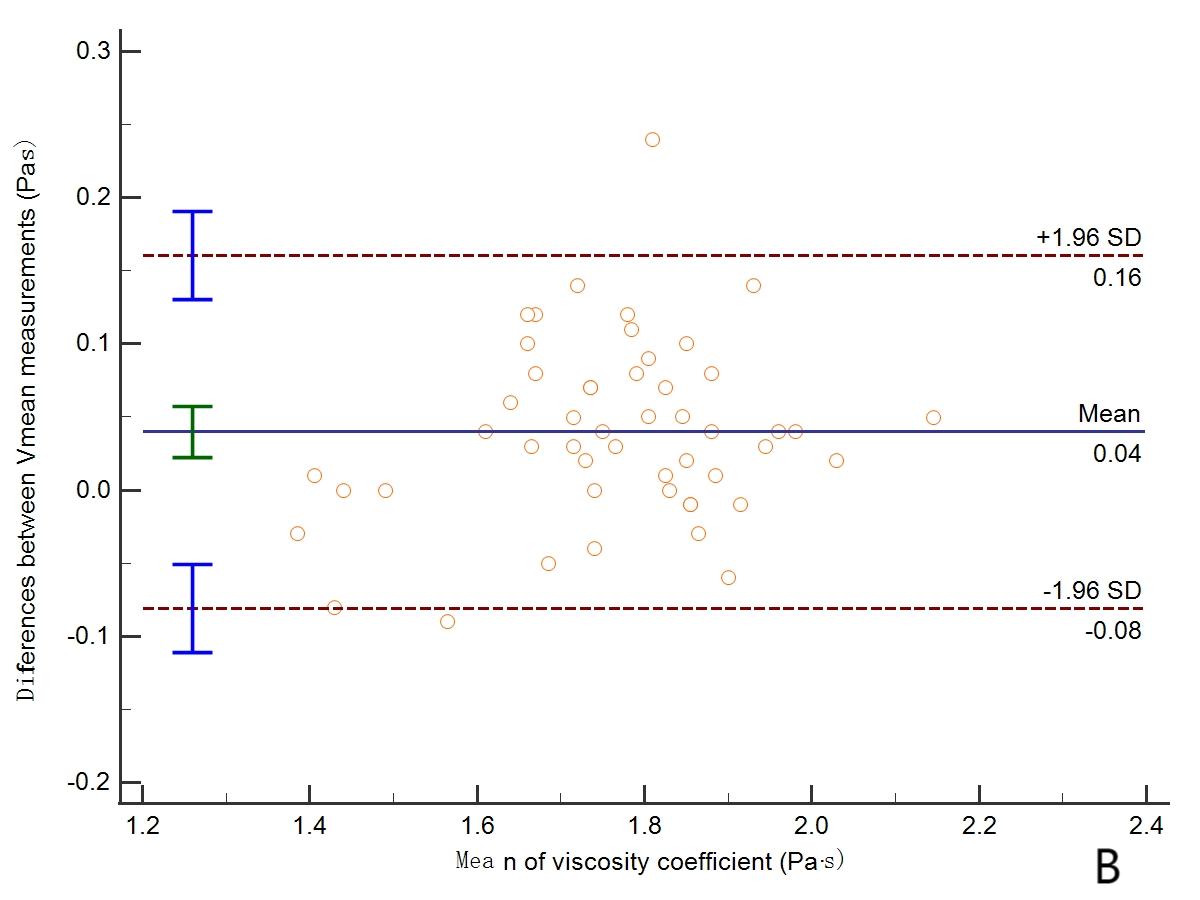


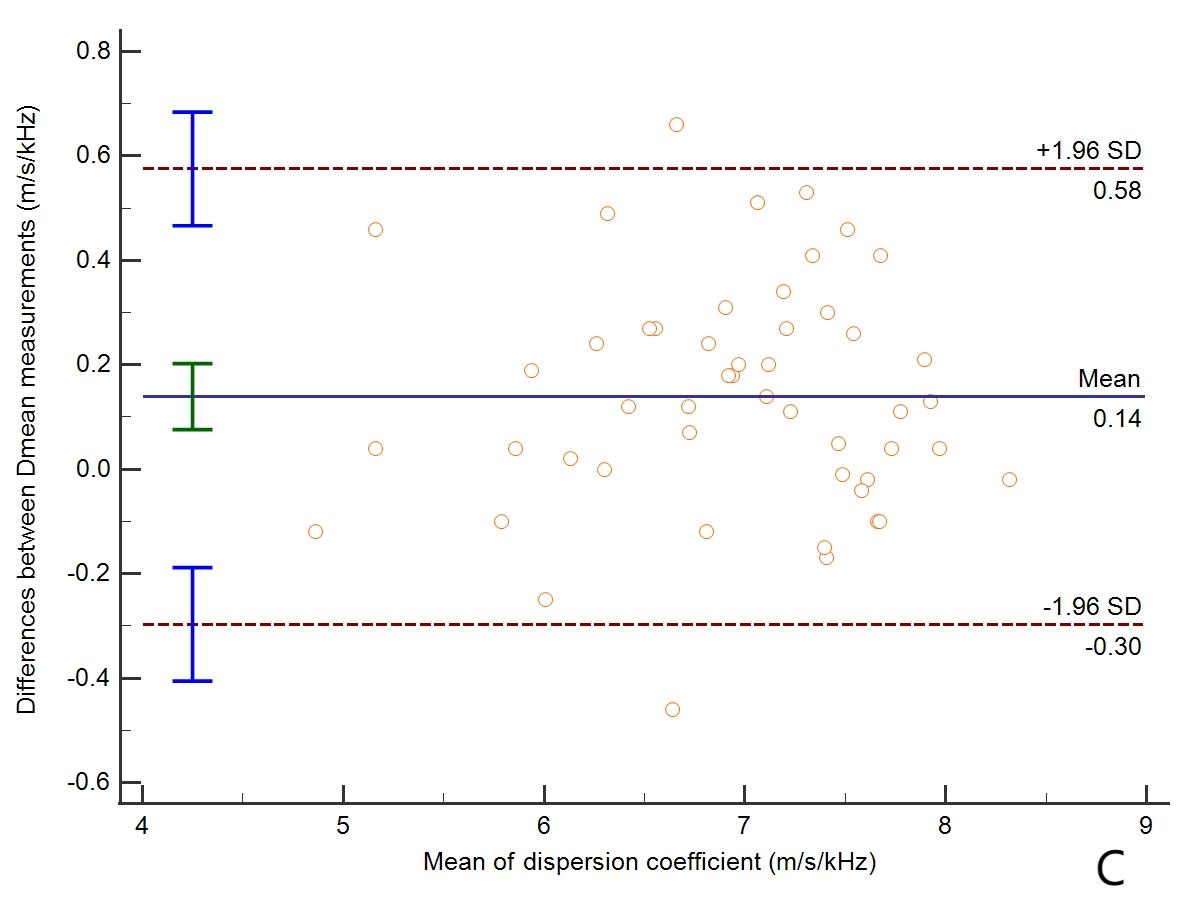


**Supplementary figure 2** Bland-Altman plot of inter-observer consistency. Horizontal dashed lines indicate 95% upper and lower limits of agreement and the mean in the middle.

（A）Bland-Altman plot of variability for Emean measurements. （B）Bland-Altman plot of variability for Vmean measurements. （C）Bland-Altman plot of variability for Dmean measurements.
